# The Insurgence of Tramadol Abuse among the Most Active Population in Jirapa Municipality: A Study to Assess the Magnitude of the Abuse and Its Contributory Factors

**DOI:** 10.1155/2021/3026983

**Published:** 2021-02-05

**Authors:** Ferguson Saapiire, Godfrey Namillah, Vincent Tanye, Abdulai Abubakari

**Affiliations:** ^1^St. Joseph Nursing Training College, Ministry of Health, P.O. Box 24, Jirapa, Wa, Ghana; ^2^Department of Public Health, School of Allied Health Sciences, University for Development Studies, P.O. Box 1883, Tamale, Ghana

## Abstract

**Background:**

Tramadol has gained popularity among the drugs of the most active population especially the respondents in Ghana abuse especially farmers who nicknamed as “farm and buy cow.” It has recently become a public health concern, and stakeholders are worried about tramadol abuse and its implications on health in the Upper West Region. The study sought to measure the prevalence of tramadol/related substance abuse and the associated factors.

**Methods:**

A community-based analytic cross-sectional study involving 420 respondents was conducted. The participants were selected using a multistage sampling technique. Semistructured questionnaire was used to generate the data.

**Results:**

About 77.6% of the respondents abuse tramadol while 83.9% of the participants take at least one other related substance or drug. Participants with history of any substance abuse were 5 times more likely to abuse tramadol [AOR = 5.15; 95% CI (1.501-17.656); *p* = 0.009], compared to respondents with no history of any substance abuse. Respondents who take tramadol to enhance sex were 4 times more likely to abuse tramadol [AOR = 3.776; 95% CI (1.352-10.545); *p* = 0.011]. Formal sector employment was protective against tramadol abuse [AOR = 0.100; 95% CI (0.017-0.595); *p* = 0.011] compared to self-employment and the unemployed. In addition, use of nonopioid prescription drugs for posttraumatic/pain management reduced the risk of tramadol abuse [AOR = 0.237; 95% CI (0088-0.640); *p* = 0.004] compared to the posttraumatic/pain management dependence on prescription of only opioid like tramadol.

**Conclusion:**

An infantile municipality like Jirapa is challenged with high level of tramadol and related substance which has serious repercussion on the health system in the Jirapa district. It is important that measures are taken by the stakeholders to stop tramadol and related substance and mitigate the impact of drug abuse in the district.

## 1. Background

The world is in opioid crisis; in fact, prescription drug abuse has monumentally increased; cocaine and opium absolutely hitting the highest records in the history of the world presenting multiple challenges on multiple fronts [1]. The extent of nonmedical use of prescription-only drugs is becoming a major threat to global public health and law enforcement worldwide. Off late, opioids have been identified to cause more harm to the world population and have accounted for 76% of deaths where drug use disorders were implicated worldwide in 2018 [1]. The new phenomenon that has been detected is that opioid use is booming as tramadol crisis emerges in Africa [2]. It is one of the most widely used drugs in several West African countries for nonmedical purposes after cannabis, which remains by far the most popular globally. Tramadol is a synthetic 4-phenyl piperidine analogue of codeine, centrally acting analgesic with strong opioid agonist properties as well as inhibitory effects on the reuptake of noradrenaline and serotonin [3]. It was initially thought to have minimal addictive potentials when compared to other opioid analgesics, but the unfolding scenarios are contrary. Drug abuse is the indiscriminate use of drugs without a doctor's prescription. This definition covers everything that people ingest, inhale, or absorb. It includes medicines, over-the-counter drugs, illegal drugs, beverages, cigarette, food additives, industrial chemicals, and even food. Drug abuse is taking of drugs to the extent that they cause social or medical harm to the take [4]. Reasons advance for drug abuse are peer group influence, psychological or physiological factors, environmental factors, unemployment, unmet expectations, and media advertisement [5]. The consequences of drug abuse include mental illness, liver and kidney-related diseases, low productivity, child abuse, sexual abuse, loss of individual integrity, financial mismanagement, destruction of family hope, and untimely death [6].

The current trend of substance abuse among women is a major national concern. It has detrimental effects on women's health and behavior and could lead to death [1].

The extent of abuse of tramadol and codeine among pupils at both basic and junior high schools in the Upper West Region is alarming [7]. It is true that tramadol has gained notoriety among the drugs that the respondents widely abused in the region [8]. No wonder some farmers in the localities have nicknamed it with the name “Koo da nan hum” literally meaning farm and buy cow [9]. Research found that students take the drug to exert their strength when having sexual intercourse with their partners while others take it just for pleasure [9]. While health workers, parents, food and drug regulators, civil society organizations, and the security agencies are all worried about the abuse of tramadol in the region, it is shown that there are even other dangerous substances which are more hazardous than tramadol that people in the district indulge in [9]. The current state of tramadol abuse is not only a threat to public health of the municipality but also a recipe for social vices or the falling standards of morality and education in the municipality. Regardless of the level of pervasiveness of the abuse, documented evidences are not available to justify the situation in the municipality. The study therefore is aimed at assessing the prevalence of the tramadol abuse and associated factors.

Tramadol is sometimes abused alongside other drugs, which is called polydrug use. Typically, users combine tramadol with other substances to increase their high or self-medicate. The following drugs are commonly combined with tramadol: alcohol, other painkillers, sedatives, like benzodiazepines and sleeping pills, and cold medicine [10].

## 2. Methods

### 2.1. Study Area

The study was conducted specifically in the Jirapa Municipality of the Upper West Region with a territorial size of 1,188.6 square kilometers. The municipality is divided into submunicipals for effective health services delivery purpose. There are seven (7) submunicipals administratively managed by Submunicipal Health Teams (SMHTs). The study took place in Hain, Tizza, Duori, Tuggo, Yaga, Sabuli, and Jirapa urban submunicipals. In 2017, the entire population of the municipality is estimated to be 101,899. The LI 1902 established Jirapa District which was carved out of the then Jirapa-Lambussie District in light of Ghana's decentralization processes in 2007 and was upgraded into a municipal status in 2018.

### 2.2. Study Design and Target Population

The study was an analytical community-based cross-sectional design involving the most active population within the ages 15-55 years. All persons below 15 years and above 55 years were excluded. All critical ill-patient was equally excluded. Persons who currently abuse/misuse tramadol were included in the study.

### 2.3. Sample Size and Sampling

Sample size was estimated using the Cochran's formula for quantitative continuous data. In addition to the 10% nonresponder rate, a sample size of 420 persons was used for the study. A prevalence of 50% was used for the estimation of the sample size with an acceptable margin of error of 5%. The respondents were selected across the seven submunicipals for the study.

A multistage sampling procedure was used for the study. Stratified sampling was used to stratify the seven submunicipals into stratums. Simple random sampling procedure was used to select the communities within each stratum for the study. This was randomly done using the ENA sampling software. A comprehensive list of all households that constituted the sample frame was compiled from a chosen cluster, and systematic sampling technique was used to select the study households. The first household was selected using the table of random numbers. In the selection of the study participants, only one eligible respondent was selected using a simple random sampling technique in a household.

### 2.4. Data Collection Methods

A semistructured questionnaire made of both open and closed-ended questions was used to collect the data from the respondents. The questionnaires were personally administered in a face-to-face interview by research assistants. The tool was to enable us to collect both qualitative and quantitative data. In addition, it enables us to collect large range of different responses from respondents. Also, the choice was made for purposes of data triangulation of responses. The questionnaire was the main instrument used for the collection of data. There were some open-ended questions as well as closed or multichoice questions. The open-ended questions provided in-depth understanding into some of the reasons underpinning the use of tramadol and other abusive substances. Face to face interview was administered because other modes (telephone and social media) would have been problematic because abusers of tramadol would have been suspicious as it is illegal to take it in Ghana.

Ample time were given to the respondents to study the pattern of the instruments and to answer appropriately without being rushed. Respondents' preferred choices were ticked. However, in instances where respondents cannot read and write, the data collector read out to the respondent in a language (Dagaare) that he/she understood. Piloting of the questionnaire was done a day after the practical training sessions of the enumerators. The team reconvened to discuss thoroughly about the entire exercise, and some misconceptions, flexibility issues of the data collection tool, and interpretations were further clarified. Also, the validity and reliability tests were ran to determine precision level of the tool. The determination was based on Cronbach's alpha. Some closed-ended questions were modified, and some were deleted until *R* = 0.8.

Data on sociodemographic and economic variables (gender, religion, occupation, education, and marital status), peer pressure, weak enforcement of regulations, posttraumatic/management dependence, curiosity, psychological/mental health challenges, and ignorance were collected. Also, data on the knowledge of respondents on the effects of tramadol abuse, the perceptions of the study population on the associated benefits of tramadol use, and the abuse of other related substances/drug were taken. In addition, data on other variables like awareness of the availability of the drugs, taking the drug with or without medical officer approval, duration, and dosages of tramadol mostly patronized were collected. A rating scale was used to assess the level of perception on the benefits associated with tramadol use and the knowledge on the consequences of tramadol abuse. The rating scale sought to ascertain the degree of acceptance or rejection to some parameters associated or related with tramadol among the study group. A scale of agree, strongly agree, do not know, disagree, and strongly disagree was used for the assessment.

### 2.5. Data Analysis

Statistical Package for the Social Sciences (SPSS) Version 21(SSPS Inc. Chicago, IL, USA) was used for the data entry, cleaning, and analysis. Missing data and wrong entries were checked, and all irregularities were corrected. The abuse/misuse of the tramadol by this study was defined as inappropriate use of the drug or use of the drug without physician's approval. The approved tramadol dosage strengths for use in Ghana by the FDA are 50 mg and 100 mg in tablets and capsules and 50 mg/ml-2 ml in injections, and therefore any intake above these strengths were considered as abuse.

Results from the rating scale assessment were statistically transformed into dichotomous variables of yes/no. Agree and strongly agree were considered as accepted (yes) while disagree and strongly disagree were considered as rejection (no) to a particular parameter. Knowledge on the effects/consequences of tramadol was a composite indicator of the study participants who have the knowledge that its abuse/misuse has an effect on a person and the number of the study group who are knowledgeable that the abuse/misuse of the tramadol can lead to mental problems including depression, insomnia, and addiction, while the knowledge on the consequences of tramadol abuse was classified as low or high. In addition, the perception of the respondents on the benefits associated with the abuse/misuse of tramadol was categorized as having low or high perception on the benefits. It was also a composite indicator. Tramadol abuse was the main dependent variable.

Descriptive statistics were generated from the data. Chi-square test was used for the bivariate analysis to establish the relationship between tramadol abuse and independent variables. A relationship was considered significant when *p* < 0.5. Multivariate analysis was done to control for confounders and to exactly identify determinants/predictors of the tramadol abuse among the study population by using logistic regression analysis. All the independent variables that proved statistically significant (*p* < *α* value) at the bivariate level were put for multivariate analysis. Socioeconomic and demographic variables (sex, religion, employment status, and educational level), knowledge on the consequences of tramadol abuse (level of knowledge on consequences, know whether tramadol abuse has an adverse effect, and know whether tramadol abuse leads to mental problems), perceptions on benefits of tramadol (level of perception on benefits, sex enhancer, and good euphoria), and other variables (reasons for the use of the drug, abuse of other related substances, posttraumatic/management dependence, and know a person abusing the drug) with *p* values less than 0.05 were considered for the modeling.

## 3. Results

### 3.1. Socioeconomic and Demographic Characteristics

The mean age of the study participants was 28.3 ± 7 years. Most of the study participants (40.7%) were found in the age group 18-25years. About 81.4% of the respondents were males. Majority of the respondents (specify the figure) were self-employed. Most participants 191 (45.5%) had at least secondary education. About 46.7% of the participants were married whereas the majority (53.3) were single. Again, most participants (67.6%) were Christians (Table 1).

### 3.2. Prevalence of Tramadol Misuse/Abuse

The prevalence of tramadol use is 36.2% among respondents in the municipality with 77.6% of the users inappropriately taking or misusing/abusing the drug. Averagely, the daily milligram intake of tramadol was 100 mg ± 42.6 mg. About 32.9% of the participants misuse tramadol without knowing the various strength/dosages they take. Regardless of the strength, 17.1% of the study participants can take at least 4 tablets/capsules at once. The vast majority of the respondents patronize dosages ≥ 100 mg (Table 2).

### 3.3. Reasons for the Intake/Misuse of Tramadol

The first three commonest reasons why majority of respondents took tramadol were peer influence (38.8%), improve physical performance (37.5%), and improve physical strength/become more active (24.3%).

**Figure 1:**
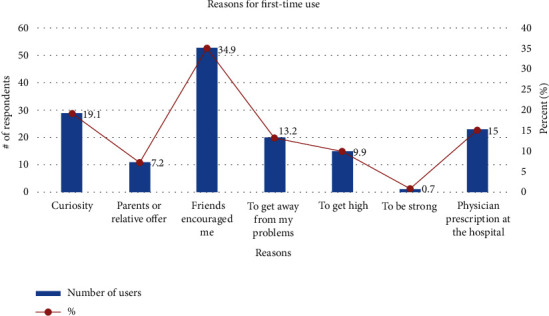
Purpose for the intake of tramadol for the first time.

Summary of the reasons indicate that majority (54.6%) took the drug to solve a single challenge while 29% use the drugs for multiple reasons. However, a significant number has no reason for the use of the drug (Table 3). The influenced by friends was the most reason (34.9%) for the first-time tramadol use and the continuous use/misuse while 19.1% of the first-time users said they took tramadol because of curiosity (Figure 1).

### 3.4. Sources of Tramadol in the Jirapa Municipality

Also, about 95.7% of the study participants were aware of the existence of tramadol in the municipality. Majority (81.5%) of the participants voted “yes” for licensed chemical shops as the main and reliable source of tramadol. Not surprising, almost 61.1% of the respondents took drug peddlers as the second most reliable source, whereas 36.7% of respondents voted “yes” for black markets as the third reliable source of tramadol for the respondents (Table 4). The remaining 22.2% and 17.3% of respondents voted for hospital and moving vans, respectively.

### 3.5. Prevalence of Other Substances Abuse by the Respondents in the Municipality

About 64.5% abuse alcohol, and almost half of the respondents inhale substances popularly known as sera or Enye or snuff. Also, 17.9% and 6.4% were found abusing prohibited substances like cocaine/heroine and wee/marijuana, respectively. Interestingly, quite a number of respondents were engaged in abusing emerging substances such as “Wuole” (17.1%), sniffing glue (6.2%), inhaling petrol/turpentine (1.9%), and drinking of soaked/boiled diapers/pads (1.4%) (Table 5).

About 38% and 34% of the abusers attained at least secondary education and basic education, respectively. The remaining 28.8% had no formal education (Table 6). A positive association (*p* = 0.046) was observed between the educational status of the participants and abuse of tramadol. Compared to the unemployed (39.9%) and respondents engaged in formal employment (2.5%), majority (57.6%) of abusers were self-employed. However, the employment status of the respondents was significantly associated with tramadol abuse (*p* = 0.003). A statistically significant positive association also was observed between religion and tramadol abuse. In spite of this, about 59.4% of the abusers were Christian. Finally, it was gender/sex showed a strong positive association with tramadol abuse (*p* = 0.009).

A higher proportion (63.2%) of the respondents believed that there are extraordinary benefits associated with abusing tramadol. Therefore, the perception of respondents on the benefits of tramadol use was significantly related to its abuse (*p* = 0.027). Also, taking tramadol for purposes of enhancing sexual performance/prolongation of the time of intercourse (*p* = 0.001) and taking tramadol to improve on euphoria/pleasurable effect (*p* = 0.046) were statistically significantly associated with tramadol abuse. It was observed that quite majority (65.1%) did not have adequate knowledge on the dangers/consequences associated with tramadol abuse. However, there was a positive association (*p* = 0.009) between tramadol abuse and the knowledge index on dangers.

Furthermore, approximately 89.5% of the respondents took the drug to relieve pain. In addition, knowledge of a person taking tramadol without a physician prescription was associated with a person abusing tramadol (*p* = 0.001). It was noted that almost 74.3% of the tramadol users have a friend or a relative abusing tramadol. Though a few (38.2%) assented to role of posttraumatic/pain management dependence in tramadol abuse, a positive association was observed between them (*p* = 0.044). A lot of (77.6%) of the respondents were involved in other related substance abuse. A positive significant association was also observed between history of other substance abuse and tramadol abuse (*p* = 0.001) which were relatively linked to tramadol abuse. Close to 78.9% have justified reasons for the abuse of tramadol (Table 7).

### 3.6. Determinants of Tramadol Abuse among the Most Active Population

The results of the logistic regression revealed that the occupation status, abusing tramadol for purposes of sexual enhancement, a history of other substance abuse, and posttraumatic/pain management dependence were statistically significantly associated with the abuse of tramadol.

This set of variables accounted for 30.5% of the variability of abuse/misuse of tramadol (Nagelkerke *R* Square = 0.305). Compared to respondents with no history of any substance/drugs abuse, respondents with history of any substance abuse were 5 times more likely to be engaged in the misuse/abuse of tramadol [AOR = 5.15; 95% CI (1.501-17.656); *p* = 0.009]. The odds of abusing tramadol for purposes of sexual enhancement was higher among respondents. Compared to respondents who rejected the perception that tramadol was a sex enhancer, the respondents who take tramadol to enhance sex were 4 times more likely to take tramadol without physician prescription/inappropriately engaged in taking tramadol [AOR = 3.776; 95% CI (1.352-10.545); *p* = 0.011]. The odds of respondents abusing tramadol was significantly higher among unemployed respondents [AOR = 0.100; 95% CI (0.017-0.595); *p* = 0.011] compared to respondents who were engaged in government work or self-employed. Comparatively, there is an increased risk of 23.7% of the respondents likely to continuously depend on opioid prescription-only drugs like tramadol after been used to manage trauma or pain-related illness in the hospital [AOR = 0.237; 95% CI (0088-0.640); *p* = 0.004].

The rest of the independent contributory variables were statistically not significant (*p* ≥ 0.05) and were removed by hideout from the model during the modeling process (Table 8).

## 4. Discussion

### 4.1. Prevalence of Tramadol Abuse, Clinical and Drug Related Parameters and Other Related Substance Abuse

The current study identified that 402 (95.9%) and 280 (66.7%) of the respondents had heard and saw tramadol, respectively. Out of this number 152 (36.9%) and 118 (77.6) had individually used and abused/misused tramadol. Elhabiby in his systematic analysis found similar findings especially in many African countries [11]. For example, a prevalence of 64% abuse of tramadol was reported in Nigeria, which was consistent with the findings of the present study [11]. In the united State of America, the number of tramadol abuse or misuse increased approximately 250% from 2005 to 2011 among visitors of the emergency department [12]. The World Health Organization (WHO) warned the growing evidence of tramadol abuse in many African and West Asian countries despite the challenge of large seizures of such preparations in North and West Africa [13]. Also, a study by Elliason et al. made the same revelation about the level of awareness and knowledge of the respondents on the availability of tramadol [8].

The abuse of substances and other drugs in the municipality is alarming as unearthed by the study. The emergence of new forms of abuses puts the municipality at risk of public health disasters and rising trends of social vices. The called by the District Director of Health Services of Sunyani West district in the Brong-Ahafo region of Ghana reaffirmed the findings of the current study about the looming danger of abuse of “Wuole” (a mixture of akpeteshie or other jin and wee or cocaine with or without ginger) in Ghana [14]. About 6.2% of the respondents were found sniffing glue despite the adverse consequences associated with it. This abuse is widespread across the world. A study in the USA has reported that nearly 20% of adolescents have experimented with illicit drugs. Sniffing among other inhalants constitutes the first drugs that are being experimented by the adolescents [15]. Also, other emerging substances like drinking of soaked/boiled diapers/pads, inhaling of petrol/turpentine, and smoking of dried faeces and nim tree in the municipality are of a big worrisome situation. The surge of this new phenomenon seems to catch the eyes of most West African leaders [16].

On high prevalence of substance abuse, almost half the respondents were found abusing alcohol, Indian herm or wee, mahogany/bitter roots with alcohol/akpeteshie, and “sera” or “Enye.” In this regard, the findings of the following studies are consistent with findings of the current study [17–20]. The high patronage of sera or snuff revealed by the current study is alarming with regard to the number of respondents involved. However, findings of studies in Ghana [8, 21] are in consonance with the 45% prevalence of the respondents abusing snuff/sera as found in the present study.

### 4.2. Demographic and Socioeconomic Variables and Tramadol Abuse

The present study established that gender, religion, education status, and employment status were significantly related with the abuse of tramadol. A case study of tramadol use in Lagos state, Nigeria, established the relationship between socioeconomics, demographic characteristics, and tramadol abuse which is in support of the findings of the current study [22]. Similarly, Wassa Amenfi West Municipality study also confirmed such relationship [8]. Also, a KAP (knowledge, attitude, and practice) study by Mohammed et al. in Gaza among clients patronizing private psychiatric clinics also observed an association between socioeconomic factors and tramadol abuse [23]. A study in north-eastern Nigeria found over 93% of tramadol users being males [24]. Also, education level, gender, knowing a parson abusing the drug, and history of smoking were found related with tramadol abuse by a study in Iran which is consistent with the findings of the present study [25].

### 4.3. Perceptions on the Benefits of Tramadol, Other Reasons and Abuse/Misuse of Tramadol

The present study found that having low or high perception on the benefits of tramadol influences the abuse of the drug. The perception that the use of tramadol is a sexual enhancer and improves euphoria was significantly related with tramadol abuse. Ghana drugs report in 2019 noted that tramadol can induce a sense of euphoria and enhance sexual prowess [26]. The respondents habitually remain in the practice for various psychological and physical gratifications which include euphoria, attentiveness, high energy levels, pain relief, and improved sexual performance [27]. However, some of the actions and inactions of the youth in Ghana are attributed to the associated benefits of tramadol abuse. The Food and Drug Authority (FDA) admits that there has been reported incidence of addiction, armed robbery, youth vandalism, car accidents, and, in some cases, violence which were linked to the influence of unapproved intake of tramadol by perpetrators [28]. In addition, Delase of FDA remarked that people especially respondents take tramadol for extra energy for manual workers, euphoria (tramadol can produce euphoria comparable to heroin even at a single dose of 75 mg), and staying alert for long hours, especially for commercial vehicle drivers and their mate(s) and students, making them dazed and easily drift to deep restful sleep were some of the related benefits for the abuse of tramadol among the respondents [28]. Whereas majority of tramadol users do not intend to take the drug for a prolonged period of time, the therapeutic effects together with the feelings of euphoria and decreased anxiety often lead a user to take more than prescribed, for a longer period than originally intended (often without the prescribing physician's knowledge) [22]. It indeed a reality that tramadol has been found to significantly improve sexual satisfaction and delayed ejaculation [29]. There is widespread evidence with its ability in reuptaking of norepinephrine and serotonin in the body but delays in ejaculation is unclear [29]. A significant positive association was found between having inadequate knowledge on adverse effects and tramadol abuse. There is relatedness and consistency with the study findings in Gaza in 2013 [23]. Also, a study on adolescence abuse of the drug revealed the limited knowledge of participants on the consequences of the tramadol abuse [30]. Lastly, a significantly positive association between posttraumatic/pain management and tramadol abuse was observed. This observation is similar to the finding of a study conducted in Northern Ghana [27].

### 4.4. Predictors of Tramadol Abuse

The main findings indicated that the history of abuse of other substances, sexual enhancement, postdependence from trauma/pain management, and occupation were associated negatively with the respondents abusing tramadol. This is consistent with a study conducted in the Western Region of Ghana [8]. A study conducted in Sweden affirmed the link between the use of tramadol as aphrodisiac and been tested positive for tramadol [31]. Also, the role of postpain/traumatic dependence on the abuse of tramadol was elucidated in a critical review report [32].

Despite the addiction potential of this drug coupled with the adverse effects, the veracity of its use in the management of trauma and patients in severest pains cannot be underestimated [33]. The findings from the municipality alone with its unique culture and the inherit constraint of the study design might limit the generalizability of our findings.

In view of the study outcomes, further larger and longitudinal studies are proposed. More biochemical analyses are needed especially the constituents of the emerging substances that attract the respondents. We will recommend more vigorous investigation on substance use patterns and the physical and psychiatric comorbidity among the respondents using these substances including prescription-only opioids like tramadol.

## 5. Conclusion

The findings of the study revealed that, among the respondents in the municipality, 77.6% of the tramadol users are inappropriately taking or misusing/abusing the drug. In fact, history of abuse of other substances, sexual enhancement, postdependence from trauma/pain management, and occupation were the independent predictors of the tramadol abuse. Also, there is a looming danger since majority of the respondents are indulged in the abuse of other substances and drugs like codeine, alcohol, and snuff/sera. The trend of the insurgence of abuse of emerging substances like the smoking of dried faeces and drinking of soaked diapers/used pads is a worrisome situation and a wake-up call to stakeholders especially the law-enforcement authorities within the municipality.

## Figures and Tables

**Table 1:** Demographic characteristics.

Characteristics	Mean age (*x* ± *s*)	Frequency (*n* = 420)	Percent (% = 100)
Age	28.3 ± 7.87		
<18 yrs		9	2.1
18-25 yrs		171	40.7
26-30 yrs		113	26.9
30 yrs-55 yrs		127	30.3
Educational level			
No education		86	20.5
Basic level		143	34.0
At least 2nd cycle		191	45.5
Marital status			
Single		224	53.3
Married		196	46.7
Occupation			
Unemployed		165	39.3
Agric/galamsey		114	27.1
Artisans/hairdresser/seamstress/traders etc.		110	26.2
Govt worker		31	7.4
Sex			
Male		342	81.4
Female		78	18.6
Religion			
Christian		284	67.6
Moslem		75	17.9
Traditionalist		61	14.5

**Table 2:** Prevalence of tramadol misuse/abuse.

Characteristics	Frequency (*N* = 420)	Percent (%)	(*x* ± *s*)
Aware of tramadol	402	95.7	
Seen tramadol	280	66.7	
Tramadol use	152	36.2	
Used tramadol without physician approval/inappropriate use	118	77.6	
Duration of tramadol usage			
<1 month	17	11.2	
Less than 1 yr	32	21.1	
1-5 yrs	79	52.0	
5-10 yrs	24	15.7	
# of capsule/tablet taken daily			
One	26	17.1	
Two	72	47.4	
Three	28	18.4	
At least four	26	17.1	
Mean milligram daily intake (*x* ± *s*)			100 ± 42.6
Milligram taking on daily			
50 mg	28	18.4	
100 mg	46	30.3	
125 mg	18	11.8	
200 mg	10	6.6	
Do not know	50	32.9	
Know a person sick/mentally challenge due to tramadol abuse			
No	347	85.5	
Yes	59	14.5	

**Table 3:** Reasons for the intake/misuse of tramadol.

Reasons	Number of users (*N*)	Percent (%)
Reasons for the current use of tramadol		
Took tramadol because of sickness	28	18.4
To do more hard/physical work	57	37.5
To enhance sleep	12	7.9
To look strong and young	37	24.3
No reason	18	11.8
I do not know	5	3.3
To enhance sex	13	8.6
To release stress	26	17.1
Involved in road traffic accident	8	5.3
Parents or relative offer	11	7.2
Friends encouraged me	59	38.8
To get away from my problems	21	13.8
To get high (good euphoria)	22	14.5
Prescription given by a physician	23	15.1
Summary of reasons of the use of drugs		
No reason at all for the use of drug	25	16.4
Single reason	83	54.6
Multiple reasons	44	29.0

**Table 4:** Sources of tramadol.

Sources	# of participants of the study who agreed to the following sources of tramadol	%
Licensed chemical dealers	331	81.5
Drug peddlers	248	61.1
Black markets	149	36.7
Hospital	90	22.2
Moving vans	70	17.3

**Table 5:** Prevalence of other substance abuse.

Substance	# of abusers *N* = 420	Percent (%)
Cocaine/heroine	27	6.4
Smoking of wee/marijuana	75	17.9
Alcohol abuse	271	64.5
Sniffing of snuff (“sera”/“Enye”)	189	45.0
Intake of “Wuole”	72	17.1
Smoking of nim tree	27	6.4
Intake of mahogany roots with alcohol	132	31.4
Drinking of soaked/boiled diapers/pads	6	1.4
Inhaling of petrol/turpentine	8	1.9
Smoking of dried faeces	11	2.6
Sniffing glue	26	6.2

“Wuole” ^∗^(mixture of boiled wee and akpeteshie or local jin and with/without ginger).

**Table 6:** Sociodemographic characteristics associated with tramadol abuse.

Demo-socioeconomic characteristics	Misusers/abusers of tramadol	*p* value
	*N* (%) = 118	
Sex		
Male	107 (90.7)	0.009
Female	11 (9.3)	
Religion		
Christian	70 (59.4)	0.018
Moslem	24 (20.3)	
Traditionalist	24 (20.3)	
Age		
<18 yrs	2 (1.7)	0.270
18-25 yrs	47 (39.8)	
26-30 yrs	36 (30.5)	
30-55 yrs	33 (28.0)	
Education		
No education	34 (28.8)	0.046
Basic level	40 (33.9)	
At least 2nd cycle level	44 (37.3)	
Marital status		
Single	66 (55.9)	0.069
Married	52 (44.1)	
Occupation		
Unemployed	47 (39.9)	0.003
Self-employed	68 (57.6)	
Government worker	3 (2.5)	

**Table 7:** Other factors associated with tramadol abuse.

Reasons for tramadol abuse	Variable category(*N* = 152)		*p* value
	Yes *n*/*N* (%)	No *n*/*N* (%)	
History of any other substance abuse	133 (77.6)	19 (22.4)	*p* < 0.001
Relieve pain	136 (89.5)	16 (10.5)	0.530
Increase personal euphoria	102 (84.1)	50 (15.9)	0.046
Relieve stress/frustration	98 (64.5)	54 (35.5)	0.235
Increase physical performance	119 (62.5)	33 (37.5)	0.216
Improve sexual performance	86 (56.6)	66 (43.4)	0.001
Enhance sleep	58 (38.2)	94 (61.8)	0.696
Boost appetite	51 (33.6)	101 (66.4)	0.160
Relieve of frustration	70 (46.5)	82 (53.5)	0.069
Become courageous/brave	79 (52.0)	73 (48.0)	0.153
High perception level on benefits	96 (63.2)	56 (36.8)	0.027
Knowledge on dangers/consequences	53 (34.9)	99 (65.1)	0.009
Bad parental influence	70 (46.1)	82 (52.9)	0.894
Peer influence	127 (83.6)	27 (16.4)	0.625
Curiosity	129 (84.9)	25 (15.1)	0.642
Easy accessible/not properly regulated	93 (61.2)	59 (38.8)	0.380
Poor parental control	83 (54.6)	69 (45.4)	0.179
Posttraumatic/pain management dependence	58 (38.2)	94 (61.8)	0.044
Over-the-counter drug patronage	77 (50.7)	75 (49.3)	0.931
Know a person taking tramadol without a physician prescription	113 (74.3)	39 (25.7)	0.001
Have at least a single reason	120 (78.9)	32 (21.1)	0.001

**Table 8:** Determinants of tramadol abuse/misuse among the respondents.

Variables	*p* value	Odds ratio	95% CI
History of other substance abuse			
Abuse	0.009	5.149	1.501-17.656
No abuse	^∗^Reference		
Sexual enhancement			
Yes	0.011	3.776	1.352-10.545
No	^∗^Reference		
Occupation			
Unemployed	0.011	0.100	0.017-0.595
Self-employed	0.058	0.200	0.038-1.055
Government workers	^∗^Reference		
Posttraumatic/pain treatment dependence			
Yes	0.004	0.237	0088-0.640
No	Reference		

^∗^No.of users = 152; CI: confidence interval; ref: reference category; Omnibus test (*χ*^2^ = 38.885 and *p* = 000) and Hosmer and Lemeshow test (*p* = 0.315).

## Data Availability

Relevant data has been added in the manuscript from which all logical conclusions were driven. Do not hesitate to contact the corresponding author for the dataset.

## Abbreviations

UNODC: United Nation Office of Drugs and Crime
CYPD6: Cytochorome P450 2 D6
SMHT: Submunicipal Health Team
ENA: Essential Nutrition Action
AOR: Attributable odds ratio
CI: Confidence interval
WHO: World Health Organization
USA: United State of America
GNCB: Ghana Narcotics Control Board
KAP: Knowledge, attitude, and practice.
